# Give me five! A modern perspective on addiction from the up-dated Freudian drive theory

**DOI:** 10.3389/fpsyt.2025.1562619

**Published:** 2025-09-05

**Authors:** Michael Kirsch

**Affiliations:** University Hospital Essen, Institute of Physiological Chemistry, Essen, Germany

**Keywords:** beta-endorphin, Freudian drive disruption, surrogates mechanisms, insecure attachment, fluoxetine

## Abstract

Sigmund Freud´s drive theory, often (and erroneously) referred to as his theory of the instincts, was an early attempt to describe the motivations behind both healthy and mentally ill individuals. In his final formulation, Freud identified two main categories of drives: life drives (which he termed *Eros*), encompassing physiological drives, and a set of inherent impulses, including self-destructive tendencies (which he called “death drives”), with masochism – a form of auto-addictive disorder - as a prime example. Freud´s drive theory was developed with the framework of a 19^th^-century medical mind-set, but this model has since been up-dated in various publications using advanced data from neuroanatomy, (neuro)endocrinology and biochemistry. Modern research has shown that all physiological drives (i.e., hunger, thirst, sleep, sexual drive and attachment) are regulated in key brain regions, including the *nucleus accumbens* and *lateral hypothalamus*, by neurotransmitters like dopamine and 5-hydroxytryptamine. These, in turn, stimulate the release of β-endorphin, maintaining its levels within physiological norms. Importantly, nearly all forms of addiction are associated with altered β-endorphin levels, and one or more essential drives are disrupted. Since addictive behaviors often aim to restore β-endorphin levels to normal, addiction - whether behavioral or substance-related - acts, from the perspective of an up-dated Freudian drive theory, as a surrogate for malfunctioning drives. To restore all five healthy drives, particularly attachment, it is crucial to recognize and address the underlying causative factors, such as trauma, epigenetic changes, genetic predisposition, environmental stress, or co-occurring chronic illnesses.

## 1 Introduction

Adolescence is a critical period for the development of addictive behaviors. In the United States, the 2018 prevalence of lifetime substance use among adolescents was reported to be 26.3% for alcohol, 15.3% for cannabis, and 13.4% for tobacco (1). On one hand, substance use during adolescence can initiate the persistent non-medical use of opioids into adulthood (2). On the other hand, it can significantly disrupt brain development (3–5).

Furthermore, there is a strong comorbidity between mental health issues and substance use disorders among adolescents. For example, one study found that 40.8% of adolescents in public mental health treatment programs also met the criteria for a substance use disorder (6).

This raises the question of what drives addiction. Given that independent causal factors - such as childhood trauma and chronic illness (discussed below) - can lead to addiction, it becomes important to explore whether the driving force behind addiction is dependent or independent of these causal factors.

To address this question and optimize addiction treatment, a comprehensive theoretical construct of human motivation, strongly supported by clinical data, is needed. Such a framework is currently somewhat lacking. In this manuscript, an updated version of Freudian Drive Theory is proposed to describe all forms of addiction. This revised theory posits that all addictions stem from a deficit of a specific signaling molecule, and the addictive behavior is executed as an attempt to compensate for this deficit. Since the theory´s predictions are underpinned by experimental clinical data, the updated Freudian Drive Theory may serve as a useful tool for clinical therapists.

## 2 Materials and methods

Although this work is a narrative review, the manuscript is based on a structured and transparent literature selection process. Given the interdisciplinary nature of this review — drawing on sources from fields such as biochemistry, endocrinology, neuroanatomy, neurobiology, physiology, psychoanalysis, and psychiatry — a narrative format was chosen. This approach allows for the integration of heterogeneous concepts, theories, and data without the methodological constraints of a systematic review, which may be less suitable for synthesising foundational and conceptual material across disciplines.

In addition, the use of acronyms was deliberately minimised, as their meanings and conventions often vary significantly across the above-mentioned fields. Where acronyms were used, they were selected to align with psychiatric usage in order to maintain conceptual coherence and accessibility for clinicians and mental health professionals.

Prior to writing, a detailed chapter outline was developed to guide the organisation of the review. For each chapter, relevant literature was identified using general search engines (Google and Bing) and scientific databases (primarily Medline). When these methods did not yield sufficient or suitable sources, additional material was retrieved through manual searches in the holdings of the university library.

Artificial intelligence-based tools such as ChatGPT and Perplexity were also tested, but proved unsuitable for identifying original and citable sources in this context.

In total, approximately 1,500 references were identified. Of these, about 200 were selected and included in the final manuscript following critical reading and evaluation over a two-year period.

## 3 Freudian drive theory

### 3.1 Traditional Freudian drive theory

In his early work, Sigmund Freud proposed the existence of two primary motivational drives: the sexual drive (*libido*) and the self-preservation or ego drive (7). Later, Freud refined this theory to encompass auto-addictive conditions like masochism (8). He posited two main drive collections: life drives (called by Freud *Eros*), which included ego drives, the sexual drive and those that foster human connection) and “death drives”, which were less clearly defined but suggested as an inherent collection of impulses related to destruction, aggression, and self-harm (9, 10).

Freud´s efforts to understand the drives influencing human behavior emerged from his clinical experience with neurotic and psychotic patients. The development of his drive theory and its central theoretical postulates was shaped by both clinical observations and the scientific knowledge available to him at the time. The Freudian drive theory represents an early attempt to objectively explain human behavior using verified clinical and scientific data.

**Scheme 1:**
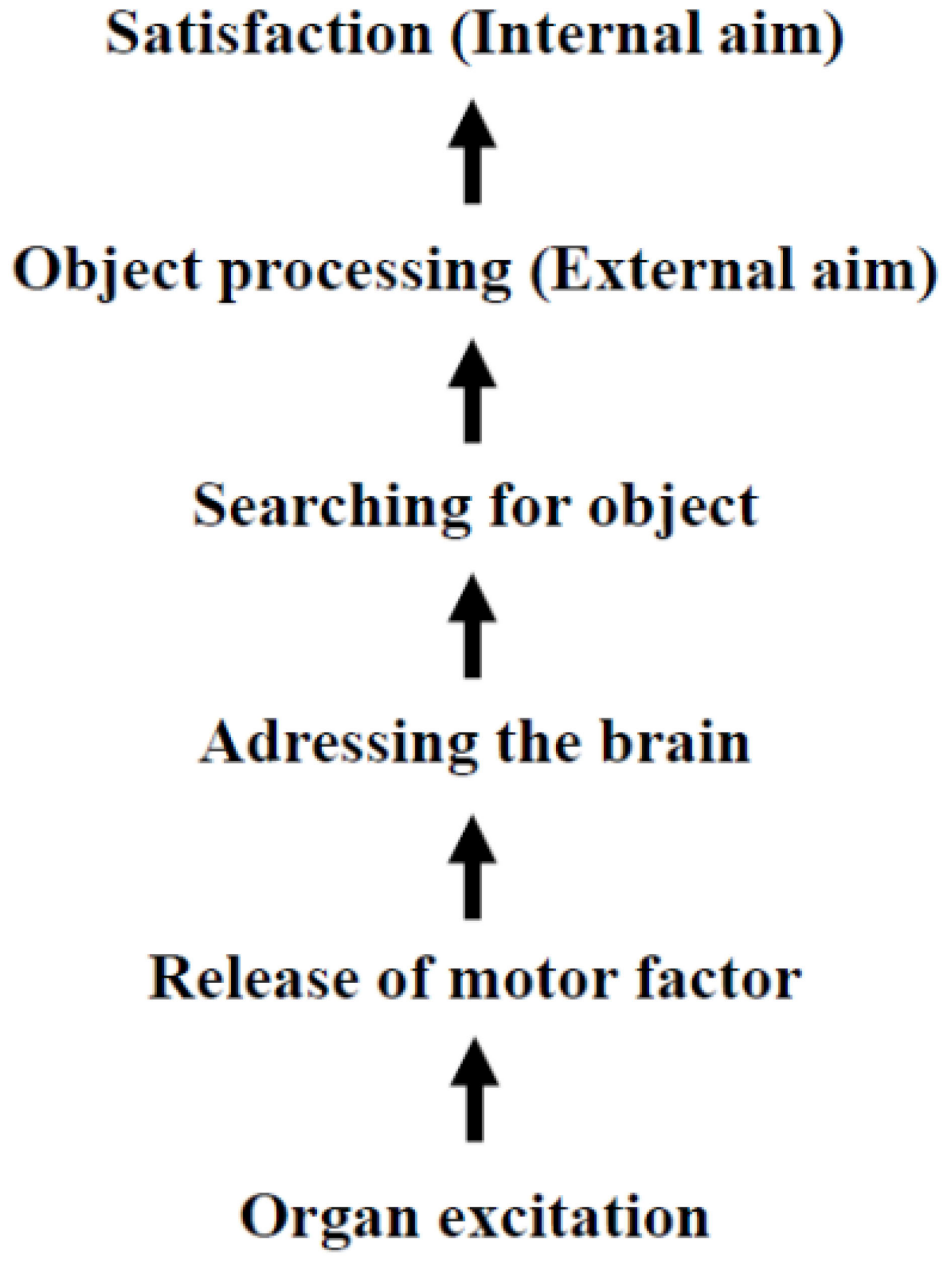
Architecture of classical Freudian drives developed between 1905-1933.

In 1905 Freud clarified the origins of drives: “*The source of an instinct is a process of excitation occurring in an organ and*…” [(7), p. 1492].^1^ He also described the motor component of the sexual drive: “*It seems probable, then, that* sp*ecial chemical substances are produce in the interstitial portion of the sex-glands; these are then taken up in the blood stream and cause particular parts of the central nervous system to be charged with sexual tension*.” [(7), p. 1530]. Freud suggested that a drive is initiated by the excitation of an organ, thereby activating the motor component of the drive, which acts as the executing entity. In 1915, Freud outlined the architecture of drives, identifying four components: pressure (or motor factor), aim, object and source ((12), p. 2960). He asserted that the “*aim [Ziel] of an instinct is in every instance satisfaction*” [((12),p. 2960]. Notably, Freud´s concept of the “aim” refers to a psychological goal (satisfaction), not necessarily the physical competition of an act – such as eating food in response to hunger. In 1933 Freud elaborated on the connection between aim and object: “*The aim can be achieved in the subject´s own body: as a rule an external object is brought in, in regard to which the instinct achieves its external aim; its internal aim invariably remains the bodily change which is felt as satisfaction*.” [(10), p. 96]. Based on these statements, the following Scheme can be constructed:

It is noteworthy that in 1905, Freud could not use the term “hormone” to describe the motor component of drives, as the term was only coined in the same year by Starling [(13), p. 340]. In subsequent years, Freud retained his original terminology to maintain consistency with his earlier writings. Since **Scheme 1** intrinsically predicts that peripheral hormones (unknown in 1905)^2^ can cross the blood brain barrier and posits the existence of an unknown metabolite that produces satisfaction as a feedback signal for human well-being, these declarations were highly speculative at the time of their presentation.

### 3.2 Fortuitously support for Freudian drive theory

Surprisingly, even scientists who did not intend to support the Freudian Drive Theory have uncovered mechanisms that align the framework outlined in **Scheme 1**. The first major contribution came from Henry´s landmark study on the physiological role of endorphins in the central nervous system (15). Henry concluded: “*Finally, when the endorphin system is hypoactive, and here perhaps duration of hypoactivity might also play an important role, an increased drive ensues to satisfy a deprived state, whether this is an appetite for food, water, social contact, sexual satisfaction, etc.:*” [(15), p. 239]. This conclusion supports Freud´s assertion that all drives share a common internal aim.

Given that various β-endorphin derivatives maintain a general state of well-being and pleasure by binding to the corresponding µ-receptor (16, 17), Freud´s concept of an internal aim (i.e., human satisfaction) can be interpreted through the release of central β-endorphin. While other endorphin families may also be involved, they are currently less well understood than β-endorphin derivatives.

The second significant insight comes from the Incentive-Sensitization Theory developed by Berridge and Robinson (18). This theory highlights the role of dopaminergic systems, not only in the sensation of satisfaction (reward^3^) but also in the process by which incentives gain motivational significance over time. The theory posits that dopamine is more closely associated with ‘wanting’ rather than ‘liking’. In other words, dopamine intensifies the pursuit and desire for satisfaction rather than the actual pleasure derived from it, which is achieved through the release of β-endorphin (as discussed above).

The third key support came from Panksepp, the principal developer of the concept of emotional Command Systems. Panksepp (21–24) proposed seven distinct types of motivations that drive specific behaviors, such as *seeking resources*, *lust*, *caregiving*, *panic*, *rage*, *fear* and *play*. These behaviors are associated with characteristic subcortical regions of the brain, collectively termed as Command Systems. For example, the brain areas associated with the motivation to *seek resources and satisfaction* are labelled as the SEEKING system.

Of particular importance to the refinement of Freudian Drive Theory is Panksepp´s concept of SEEKING, which acknowledges drives as inputs (21). This model operates on the principle that drives act as independent impulse generators ((25), p. 18). For Freud´s drive construct (**Scheme 1**) to remain valid, the hormones executing a drive must simultaneously target the brain areas characteristic of SEEKING (e.g., *nucleus accumbens* and *lateral hypothalamus*) to induce dopamine release, as well as an additional brain region specific to the particular drive in question.

An additional advantage of Panksepp’s concept of Emotional Systems is its usefulness as a framework for distinguishing motivational drives from instincts (11). The terms *drive* and *instinct* have often been used interchangeably since their inception, leading to conceptual confusion. In principle, five core drives can be identified — attachment, hunger, thirst, sexuality, and sleep — alongside four basic instincts: fear, panic, rage, and play.

Instincts are triggered by sensory input or reflexive mechanisms and are regulated by the five primary drives. Thus, a drive can activate an instinct when needed through its corresponding effector hormones, whereas an instinct cannot, in turn, activate a drive (11).

### 3.3 Purposefully support for Freudian drive theory

Over the past decade, Johnson and colleagues have provide significant evidence supporting Henry´s prediction by demonstrating that healthy individuals maintain opioids concentrations (with β-endorphin as the most effective µ-receptor activator) within a characteristic range in the central nervous system (26–31). Persistent opioid levels that are either abnormally high (as observed in certain forms of autism (26) or abnormally low (as seen in patients with *anorexia nervosa*) are indicative of pathophysiological conditions (**Table 1**).

**Table 1:** Levels of β-endorphin in disorders relative to control values.

Entry	Disorder/Condition	Disorder value in regard to control value [%]	Observed control value of β-endorphin [concentration]	Reference
1	Obesity due to hyperphagia Obesity without co-morbidity	~ 270% ~ 200%	19.0 ± 1.5 pM 3.8 ± 0.5 pM	T1_1a T1_1b
2	Autism (pubertal)	~ 260%	119.5 ± 5.3 pM	T1_2
3	Schizophrenia: chronic course episodic course	~ 170% ~ 115% ~ 188%	17.5 ± 7.2 pM 17.5 ± 7.2 pM 116.9 ± 61.7 pg/mL	T1_3a T1_3b
4	Major suicide repeaters (attempts ≥ 6)	~ 155%	116.9 ± 61.7 pg/mL	T1_3b
5	Prader-Willi-Syndrome (children)	~ 145%	402 ± 162 pg/mL	T1_4
6	Fasting of healthy men: 5^th^ day 10^th^ day	~ 140% ~ 140%	3.6 ± 0.5 pM 3.6 ± 0.5 pM	T1_5
7	Austism (postpubertal)	~ 125%	119.5 ± 5.3 pM	T1_2
8	Healthy control	100%	—–	—
9	Bulimia nervosa (female)	~ 75% ~ 75%	79.5 ± 8.5 pg/mL 98.0 ± 17.7 pg/mL	T1_6a T1_6b
10	Gambling disorder “Horse Race”: pre Race post Race	~ 65% ~ 100%	5.9 ± 3.1 pM 5.9 ± 3.1 pM	T1_7
11	NSSI pre NSSI NSSI act post NSSI	~ 65% ~ 80% ~ 130% ~ 100%	39.0 ± 19.5 pg/mL 14.3 ± 15.4 ng/mL 14.3 ± 15.4 ng/mL 14.3 ± 15.4 ng/mL	T1_8a T1_8b
12	PTSD	~ 65%	5.7 pM	T1_9
13	Chronic neuropathic pain	~ 60%	66 ± 11 pg/mL	T1_10
14	Nonpsychotic unipolar depression	~ 50%	0.42 ± 0.21 ng/mL	T1_11
15	Subtance Use Disorders Alcohol Use Disorder with comorbid depression	~ 50% ~ 80% ~ 65%	116.9 ± 61.7 pg/mL 368.2 pg/mL 368.2 pg/mL	T1_3b T1_12
16	Chronic heroin users	~ 50% ~ 35%	15.6 ± 12.4 pg/mL 1024 pg/mL	T1_13a T1_13b
17	HIV infection with chronic pain	~ 50% ~ 25%	ca. 100 pg/mL	T1_14
18	Anorexia nervosa (low weight)	~ 25% ~ 35%	ca. 6 fmol/mL 3.7 ± 2.2 fmol/mL	T1_15a T1_15b
19	Bipolar disorder type-I Manic patients Depressed patients	~ 25% ~ 30% ~ 15%	12 ± 2 pg/mL 12 ± 2 pg/mL 12 ± 2 pg/mL	T1_16

NSSI, Non-Sucidal Self Injury; PTSD, Post-Traumatic Stress Disorder, T1_1a: (32), T1_1b; (33), T1_2: (34), T1_3a: (35), T1_3b: (36), T1_4: (37), T1_5: (38), T1_6a: (39), T1_6b: (40), T1_7: (41), T1_8a: (42), T1_8b: (43), T1_9: (44), T1_10: (45), T1_11: (46), T_12: (47), T_13a: (48), T1_13b: (49), T1_14: (50), T1_15a: (51), T1_15b: (52), T1_16: (53).

### 3.4 Advances in Freudian drive theory

**Scheme 2:**
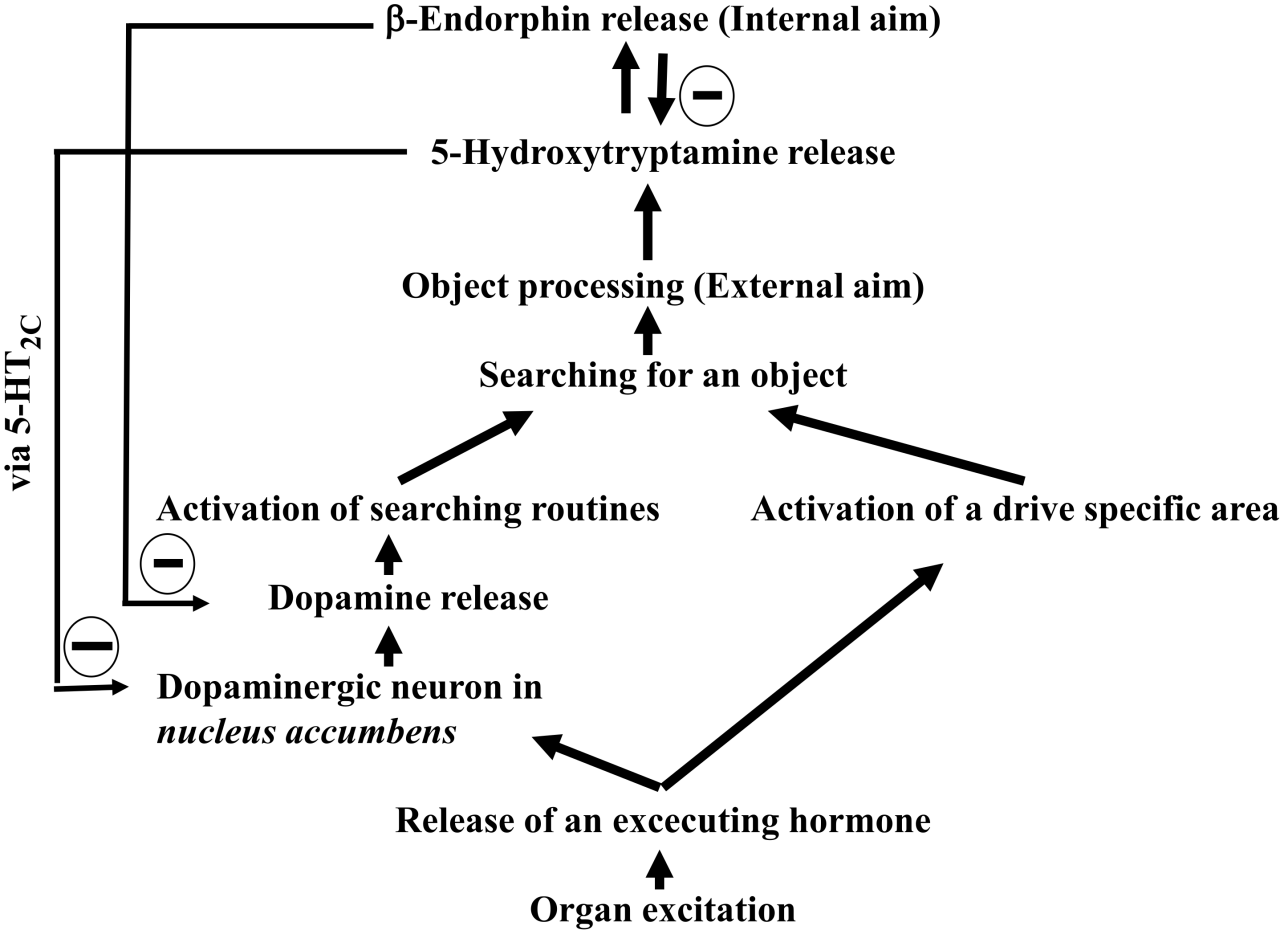
Architecture of traditional Freudian drives (updated with findings from 1982 to 2022).

The researcher of this manuscript evaluated the previously overlooked drive-specific areas, hormones and the biochemical mechanisms to further refine Freudian Drive Theory (8, 54–56). These findings culminate in an updated model, presented as **Scheme 2**:

### 3.5 Mother-infant attachment as Bowlbyian drive

**Scheme 3:**
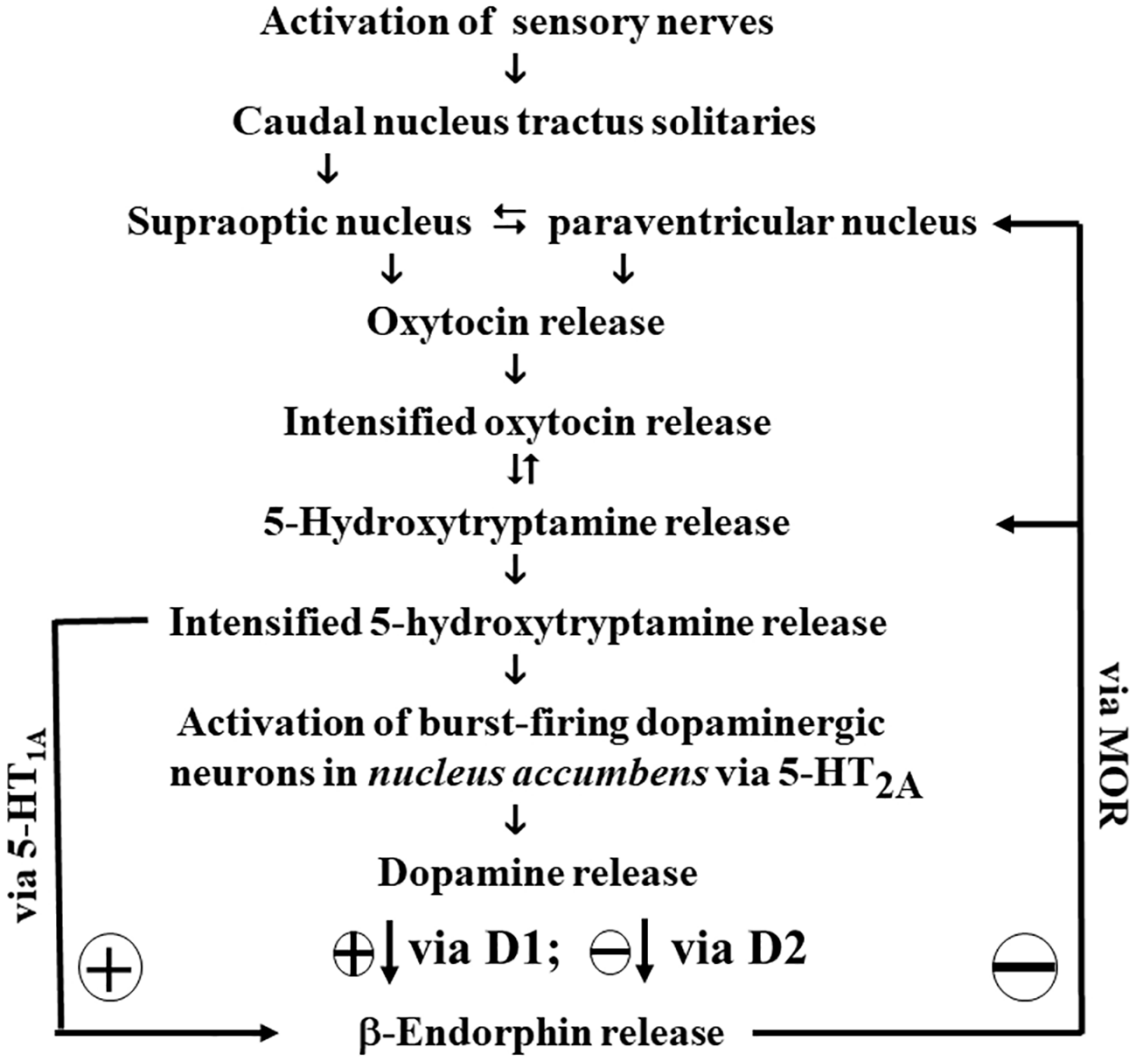
Architecture of the updated mother-infant tie (infant perspective).

While **Scheme 2** explains the onset of certain disorders, it lacks a framework for understanding complex social interactions. For instance, Harlow´s observation that attachment (e.g., in monkey infants) can be temporally more important than hunger (57) cannot be explained by **Scheme 2**. Recently, the architecture of the mother-infant tie was deconstructed from the perspective of the infant (55), and has been up-dated^4^ as shown in **Scheme 3**:

At first glance, **Scheme 3** appears structurally similar to **Scheme 2**. Due to this resemblance, the mother-infant tie is classified here as a Bowlbyian drive, honoring John Bowlby´s seminal work on attachment theory (55). Paradigmatically, the other-infant tie serves as a foundational example of attachment. However, the broader spectrum of human attachment drives (e.g., pair bonding) is likely mediated by additional signaling molecules, making the mother-infant tie a basal but comprehensive model of attachment.

## 4 Surrogates mechanisms

**Scheme 4:**
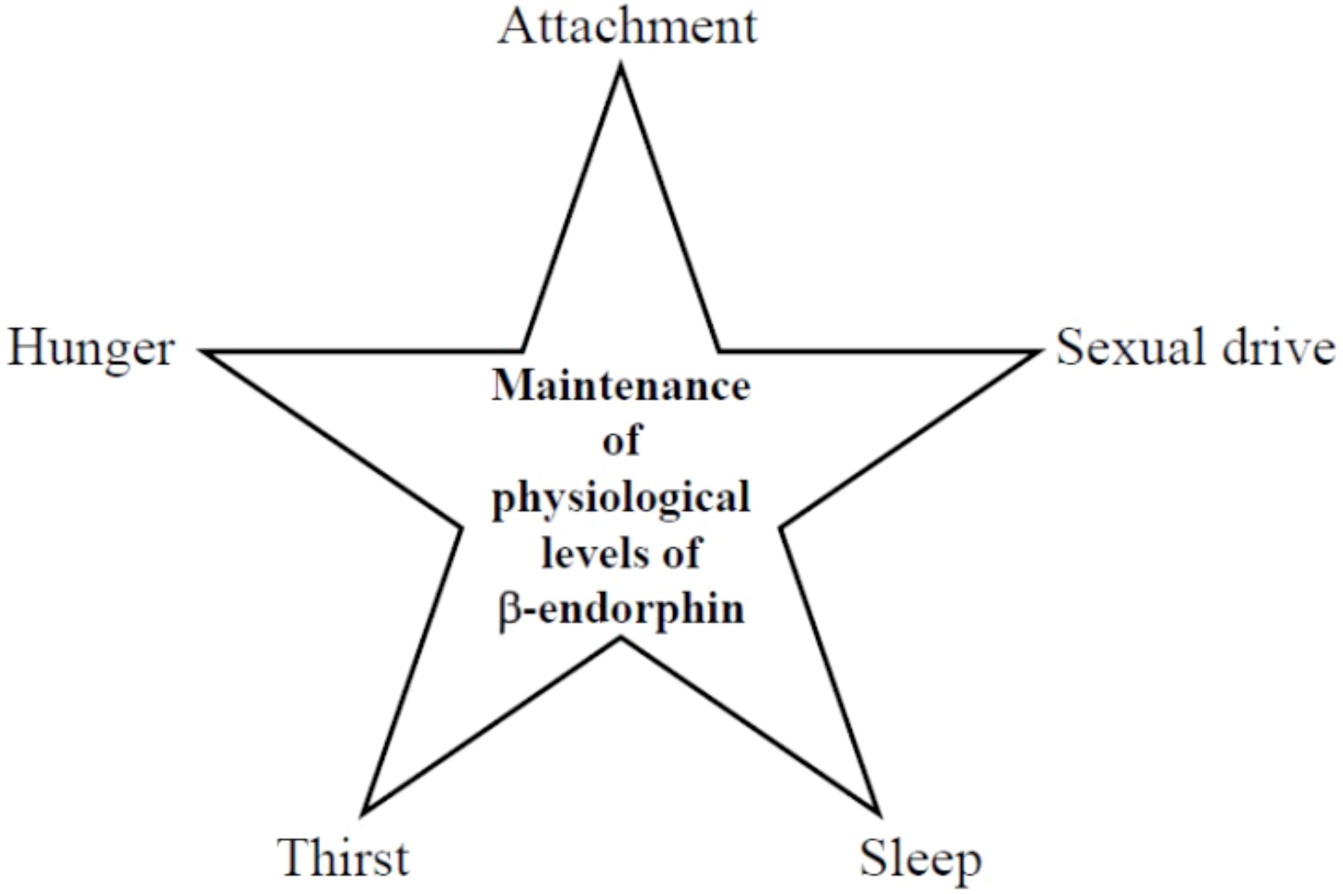
Maintenance of basal physiological β-endorphin levels by drives.

Furthermore, consistent with Henry´s earlier predictions, the attachment drive also facilitates β-endorphin release (*vide supra*). This insight allows the construction of **Scheme 4**, which emphasizes the role of basal physiological β-endorphin levels in maintaining key drives:

Thus, if one of the β-endorphin providers reduce its activity for any reason, the basal physiological β-endorphin level can no longer be maintained (examples are provided in **Table 1**, *vide supra*), and the patient unconsciously begins to seek alternative sources (8). Patients with a hypoactive drive unconsciously utilize three different strategies to compensate for the endogenously decreased β-endorphin release:

1. Overstimulating another physiological drive(s);
2. Engaging in behaviors that become compulsive and persist despite harmful consequences (often referred to as non-substance addiction or auto-addictive diseases);
3. Using drugs (substance addiction).

### 4.1 Overstimulating a physiological drive

Overstimulating the hunger drive is an effective way to release β-endorphin (**Table 1**, Entry 1). As a result, a hyperactive hunger drive is often developed as a coping strategy to counteract the hypoactive one. **Table 2** provides examples of obesity, as an excessive hunger drive, compensating for hypoactive attachment, sleep or sexual drives:

**Table 2:** Obesity as a “Coping” strategy for a hypoactive drive.

Hypoactive drive	References
Attachment due to social exclusion	(58–61)
Sleep	(62–64)
Sexual drive	(65–67)

The relationship between obesity and hypoactive drives is complex, with both factors influencing each other. For instance, obesity can induce hormonal changes that affect sexual function (68), physical limitations (69), and psychological issues such as low self-esteem, (70)). These factors further suppress the sexual drive, reducing β-endorphin release, which in turn amplifies hunger drive activity and exacerbates obesity.

In summary, when patients adopt coping strategies to maintain physiological β-endorphins levels, two drives are disrupted: one becomes hypoactive, while another becomes hyperactive, creating a feedback loop. This cycle further suppresses the hypoactive drive while intensifying the hyperactive one, perpetuating the imbalance.

### 4.2 Non-substance addiction

A childhood trauma is characterized as the experience of an extreme stressor that functions as a persistent threat to a child during early life (71). Typical examples include neglect and abuse, the death of a caregiver, exposure to violence, harrowing accidents, life-threatening illnesses, and war (72). Such early life stress can leave individuals at lifelong risk of developing non-substance addictions (**Table 3**).

**Table 3:** Childhood trauma dependent non-substance addictions.

Disorder	References
Anorexia nervosa	(73, 74)
Binge eating disorder	(74)
Body-Focused Repetitive Behaviors	(75, 76)
Bulimina nervosa	(73, 74, 77)
Compulsive Buying Disorder	(78, 79)
Compulsive Sexual Behavior	(80, 81)
Gambling disorder	(82–84)
Internet addiction	(85)
Masochism	(86–90)
Non-suicidal self-injury	(91–97)
Obesity	(98, 99)
Repetition of suicide attempts	(100–102)

It is important to note that childhood trauma is not an exclusive predictor of these disorders. For instance, the prevalence of emotional childhood abuse in non-gamblers was 7.5%, while among pathological gamblers, it rose to 19.1% (103).

A childhood trauma can activate long-lasting epigenetic mechanisms, sometimes persisting into adulthood (104–106). These epigenetic changes involve modifications in DNA methylation, histone structure, or the levels of various non-coding RNAs (A. 107). **Table 4** documented epigenetic changes associated with childhood maltreatment

**Table 4:** Childhood trauma–dependent epigenetic effects on key genes of the attachment drive.

Gene	Function	Epigenetic effect of childhood trauma	Impact on β-endorphin activity	Key refs.
*SLC6A4*	Serotonin reuptake transporter	Promoter hypermethylation → ↓ expression	↓ 5-HT_1A_ activation → ↓ β-endorphin	T5_1a T5_1b
*TPH2*	Serotonin synthesis enzyme (CNS)	Hypermethylation → ↓ TPH2 expression	↓ 5-HT_1A_ activation → ↓ β-endorphin	T5_2a T5_2b
*MAOA*	Monoamine degradation enzyme	Sex/genotype-specific methylation changes	Indirect effect via serotonin tone	T5_3a T5_3b
*HTR1A*	5-HT_1A_ receptor (inhibitory)	Promoter hypermethylation → ↓ 5-HT_1A_ expression	↓ Direct β-endorphin release	T5_4a T5_4b
*HTR2A*	5-HT_2A_ receptor (excitatory)	Promoter hypomethylation → ↑ expression	↑ Stress → ↑ HPA → altered β-endorphin	T5_5a T5_5b
*OXTR*	Oxytocin receptor	Hypermethylation → ↓ receptor expression	↓ Oxytocin modulation of β-endorphin tone	T5_6a T5_6b
*OPRM1*	µ-opioid receptor (MOR)	Hypermethylation → ↓ mRNA expression	↓ MOR density	T5_7a T5_7b
*DRD1*	D1 receptor (exitatory)	Promoter hypomethylation → ↑ D1 expression	Indirect POMC stimulation → ↑ β-endorphin	T5_8
*DRD2*	D2 receptor (inhibitory)	Promoter hypermethylation → ↓ D2 expression	↓ β-endorphin (indirectly)	T5_9a T5_9b

T5_1a: (108), T5_1b: (109), T5_2a: (110), T5_2b: (111), T5_3a: (112), T5_3b: (113), T5_4a: (114), T5_4b: (115), T5_5a: (116), T5_5b: (117), T5_6a: (118), T5_6b: (119), T5_7a: (120), T5_7b: (121), T5_8: (122), T5_9a: (123), T5_9b: (117).

Arrows indicate direction of effect: ↑ Increase / Upregulation; ↓ Decrease / Downregulation; → Leads to / Results in.

These epigenetic modifications have two major consequences:

1. Neurodevelopmental and neuroanatomical alterations:

Serotonin contributes to brain development and healthy brain function (124, 125). Childhood trauma decreases serotonin activity leading neuroanatomical changes such as altered brain plasticity, (126–130).

2. Insecure attachment:

The development of disturbed attachment disorders is strongly associated with the severity and chronicity of early trauma. According to the *DSM-5*, two primary forms are recognized:

- Reactive Attachment Disorder (RAD): Characterized by emotionally withdrawn behavior toward caregivers, limited social responsiveness, and a lack of seeking comfort.
- Disinhibited Social Engagement Disorder (DSED): Involves indiscriminate sociability, reduced fear of strangers, and overly familiar behavior with unfamiliar adults.

**Table 5** summarizes the prevalence and neurobiological correlates of disturbed attachment disorders in relation to childhood trauma.

**Table 5:** Prevalence and mechanisms of trauma-related attachment disorders.

Population	RAD prevalence	DSED prevalence	Key findings
Institutionalized children	5–22%	10–36%	Highest rates among children raised in orphanages or state care (131, 132)
Foster care children	~5–10%	~10–15%	Rates vary based on stability and age of placement (131)
General population (with maltreatment)	<1–2%	~1–2%	Rare but present in cases of chronic neglect or abuse (133)

Given that childhood trauma significantly disrupts the attachment system (as illustrated in **Table 5**), the model outlined in **Scheme 3** (*vide supra*) becomes particularly relevant.

Notably, the attachment drive appears to be impaired in both gambling disorder (134, 135) and non-suicidal self-injury (136, 137), likely due to the documented epigenetic alterations outlined in **Table 4**, which may contribute to the persistent reduction in β-endorphin levels (see **Table 1**, entries 10 and 11).

For both gambling disorder and non-suicidal self-injury, experimental data are available regarding β-endorphin levels throughout the course of the illness (41, 43). These findings provide insight into the purpose of the repetitive, harmful behaviors. The evidence suggests that such behaviors act as surrogates for the malfunctioning attachment drive, as executing the addictive act restores the persistently decreased basal β-endorphin levels to normal (**Table 1**, Entries 10 and 11).

In conclusion, early-life stress can lead to a persistent decrease in 5-hydroxytryptamine levels through epigenetic modifications. Consequently, beyond impairing brain development, it results in hypoactivity of the attachment drive. These changes disrupt the ability to maintain physiological β-endorphin levels in adulthood, thereby accelerating the onset of an alternative β-endorphin source.

### 4.3 Substance addiction

There is substantial evidence that a significant proportion - typically as high as 80% to 95% - of individuals seeking treatment for drug use (e.g., alcohol, marijuana, cocaine, amphetamine, heroin) have experienced trauma (138–140). Examination of **Table 1** (Entries 15 and 16, *vide supra*) reveals that basal β-endorphin levels are decreased in pathological drug users.

From the perspective of the up-dated Freudian drive theory, drug use can be interpreted as a surrogate mechanism for the malfunctioning attachment drive. Drugs act as alternative suppliers of β-endorphin, compensating for the deficiency caused by the impaired drive. This prediction is supported by experimental evidence, as summarized in **Table 6**.

**Table 6:** Effects of applicated drugs on the β-endorphin release.

Drug	Effect	Literature
Amphetamine	Release of β-endorphin in the NAc	(141)
β-endorphin (exogenous)	µ-Receptor agonist, similar to the application of morphine derivatives	(142, 143)
Cocaine	Release of β-endorphin in the NAc	(141, 144)
Ethanol	Release of β-endorphin in the NAc	(141, 145)
Heroin, Morphine	µ-Receptor agonist	(146)
Marijuana	Release of β-endorphin in the NAc and VTA by THC	(147)

NAc, *nucleus accumbens*; VTA, *ventral tegmental area*; THC, Δ-9-tetrahydrocannabinol; the primary psychoactive ingredient in marijuana.

This evidence highlights how various substances can act as β-endorphin modulators, effectively compensating for deficiencies linked to an impaired attachment drive. The addictive behavior thereby serves as a maladaptive coping mechanism, restoring temporarily decreased β-endorphin levels.

## 5 Causative factors

When one of the five drives outlined in **Scheme 4** is disrupted, the physiological β-endorphin level can no longer be maintained. A causative factor can render a drive hypoactive, preventing the balance of physiological β-endorphin levels. While these causative factors are not fully defined, trauma (inclusive epigenetic changes), genetic predisposition, environmental stress, and chronic illnesses are proposed as independent contributors.

### 5.1 Trauma as causative factor

When trauma, such as childhood adversity or PTSD, serves as the causative factor, it may lead to attachment disorders (**Table 5**), likely by reducing the activity of key components of the attachment drive (see **Scheme 3**) through epigenetic modifications (**Table 4**). This chronic downregulation of the attachment drive prompts patients to seek surrogates to (over)compensate the decreased β-endorphin tone. Alongside coping strategies and non-substance addictions (*vide supra*), drug use becomes a viable option for these individuals. Research has shown that substance use addicts in treatment frequently exhibit insecure attachment patterns (138, 148).

### 5.2 Environmental stress as causative factor

Environmental stress is another independent causative factor for drive disruption. Stress is defined as the perception of a threat, real or imagined, to one’s well-being (149). In principle, the activation of various stress systems enhances the organism´s ability to improve its chances for survival (150). When an individual experiences stress, known as acute stress, the sympathetic-adrenal-medullary system is activated, resulting in the release of various catecholamines (151). In addition to the well-known effects of catecholamines – such as increased heart rate and reduced blood flow to digestive systems, kidneys and skin (151) –, the catecholamine noradrenaline suppresses food intake (152). Since β-endorphin is also released in response to stress (153), the underlying mechanism warrants investigation. Notably, fasting triggers β-endorphin release in healthy individuals (**Table 1**, Entry 6). It is therefore possible that the reduced food intake during acute stress contributes to an increased β-endorphin tone.

However, when acute stress persists for extended periods or its intensity increases, the hypothalamic–pituitary–adrenal (HPA) axis, which regulates numerous functions, including metabolic and immune responses, is activated to manage the stress-response (153, 154). The initial stimulation of this axis leads to β-endorphin release (153). Hyperactivation of the HPA axis is a hallmark of chronic stress and results in the release of various hormones, including corticotropin-releasing hormone (CRH) (151). Under non-chronic stress conditions, β-endorphinergic neurons are rapidly activated by corticotropin-releasing hormone, establishing a negative feedback loop that inhibits further CRH release. However, during chronic stress, this inhibitory feedback is disrupted, resulting in sustained CRH hyperactivity. As a consequence, β-endorphin’s modulatory capacity is diminished, allowing unopposed CRH-driven stress signaling. A hyperactive CRH system can act either as an anorexigenic agent by inhibiting the synthesis of neuropeptide Y (155), or as an orexigenic factor by promoting cortisol release (151).

The sustained release of cortisol impairs the ability to fall asleep and to stay asleep in adults (156) and especially in adolescents (157, 158) resulting in a hypoactive sleep drive under chronic stress (159). The increased vulnerability to addiction during chronic stress (160) may thus serve as a compensatory response to the hypoactivity for either the hunger or sleep drive.

### 5.3 Genetic predisposition as causative factor

Genetic factors can also persistently alter drive activity. For example, Prader-Willi syndrome (PWS), caused by the absence of paternally expressed imprinted genes in the 15q11.2-q13 region of chromosome 15 (161), is characterized by disrupted sleep drive activity. Excessive daytime sleepiness is reported in nearly all PWS patients (162, 163), and sleep-disordered breathing affects 44% to 100% of patients across studies (164), with one reporting a 95% prevalence based on polysomnography (165). PWS patients likely compensates for the decreased β-endorphin tone caused by a disrupted sleep drive through hyperphagic behavior. Research shows that ghrelin, the hormone governing the hunger drive (**Table 7**), is persistently elevated in PWS patients (170, 171). The complications arising from resulting obesity are the major causes of morbidity and mortality in this population (161). Notably, the hyperphagic hunger drive, which establishes high β-endorphin levels in PWS patients (**Table 1**, Entry 5), is often describe as addiction-like behavior (172).

**Table 7:** Supporting data for **Scheme 2**.

Freudian drive	Excited organ	Hormone in command	Drive specific area	Ref.
Hunger	Stomach	Ghrelin	*Arcuate nucleus*	T2_1
Thirst	Kidney	Angiotensin II	*Subfornical organ*	T2_2
Sexual drive	Testis/ovary	Testosterone/Estrogen	*Medial preoptic area* (men)	T2_3
Sleep (NREM)	Brain	Adenosine	*Tuberomammillary nucleus*	T2_4

T2_1: ( (166), p. 1611f), T2_2: (167), T2_3: (168), T2_4: (169).

### 5.4 Chronic illness as causative factor

The forth independent causative factor for addiction is chronic illness, such as HIV. Chronic HIV infection reduces nutrient intake due to appetite loss (induced by co-occurring depression or systemic inflammation) and oral or gastrointestinal complications (e.g., *candidiasis*, *esophagitis*, *cryptosporidiosis*) (173–175). As a result, 59-84% of HIV-positive individuals experience significant weight loss (175). Likely due to the malfunctioning hunger drive, basal β-endorphin concentrations in HIV-positive patients are reduced to 50% of normal levels in pain-free individuals and 25% in those with chronic widespread pain (**Table 1**, Entry 17). This low β-endorphin tone may exacerbate the psychological stress and social stigma with HIV, driving individuals to seek satisfaction through addiction. For example, a study of 10,652 HIV-positive individuals reported a 48% prevalence of substance use disorder with specific prevalence rates of 31% for marijuana, 19% for alcohol, 13% for methamphetamine, 11% for cocaine, and 4% for opiates (176). Notably, all of these substances release β-endorphin in the *nucleus accumbens* (**Table 6**).

## 6 Discussion

### 6.1 Addiction and updated Freudian drive theory

Freud´s drive theory, developed been 1905 and 1933, lacked the benefit of later scientific discoveries, which limited his ability to pinpoint specific physiological underpinnings. For instance, Freud resorted to terms like “motor factor” and “satisfaction” because the concepts of “hormone” and “endorphins” were still in their infancy during his time. This absence of precise scientific terminology may have contributed to the undervaluation of Freudian principles in subsequent theoretical constructs. Notably, Freud´s concept of “satisfaction” as the intrinsic objective of a drive is often overlooked. He proposed that every drive ultimately seeks a uniform “internal aim” — satisfaction (**Scheme 1**).

Henry (15) suggested that various drives release endorphins. Given the association between β-endorphin and feelings of satisfaction (*vide supra*), Freud´s construct can be seen as a precursor to understanding the role of β-endorphin´s in motivation. Considering the original purpose of Freudian drive theory — to explain mental disorders — it is relevant to examine the significance of β-endorphin´s in such conditions. A review of **Table 1** reveals that many addictive disorders exhibit altered β-endorphin levels.

While Freud´s original drive theory provides invaluable insights, it also present ambiguities when viewed through a modern lens. By integrating findings from Henry (15), Johnson (28), Berridge (18), and Panksepp (21); revisiting the concept of motor factors (54, 56); and introducing β-endorphin as a biochemical equivalent for satisfaction (8, 55), it is now possible to propose an updated Freudian drive theory (illustrated in **Scheme 2**, **Table 7** and **Schemes 3**, **4**).

What, then, is the precise connection between Freudian drives and addiction? Freud stated: “The libido has the task of making the destroying instinct innocuous…” ((177), p. 163). This original statement may confuse some readers, but replacing both “*Eros*/libido” with the essential drives outlined in **Scheme 4** and “death drive or destroying instinct” (masochism being a prime example) with addiction clarifies the concept. Freud´s assertion implies that essential drives can either down-regulate or prevent the onset of addiction. Notably, in laboratory animals, exogenous β-endorphin has been shown to decrease cocaine-seeking behavior (178). Therefore, the regulation Freud suggested might be feasible if both essential drives and addictions share the same termination signal (8). Since all five drives depend on dopamine, which is decreased by β-endorphin release (**Schemes 2**, **3**), addictions must also release β-endorphin to down-regulate addictive impulses for Freud´s speculative statement to hold true. Thus, it can be concluded that the addictive act, regardless of the type of addiction, releases β-endorphin. Evidence supports this conclusion for both non-substance additions (**Table 1**, Entries 10 and 11) and drug addictions (**Table 6**).

The downside of such a mechanism, where both essential drives and addictions share the same end-product inhibitor, is that addictive acts may down-regulate essential drives. Indeed, morphine and heroin users show reduced activity in both attachment and sexual drives (179). Similarly, in the sixteenth century, de la Vega observed that cocaine “*satisfy the hungry*” (180, 181). In line with this, the cocaine´s disturbance of the sexual drive is well documented, manifesting as impotence, gynecomastia, and difficulties achieving orgasm in both genders (182).

On the other hand, when a patient selects a hyperactive hunger drive as a surrogate for a malfunctioning drive, the β-endorphin deficit caused by the hypoactive drive is often overcompensated. This can result in excessively high β-endorphin levels, as observed in Prader-Willi Syndrome patients (**Table 1**, Entry 5).

**Scheme 5:**
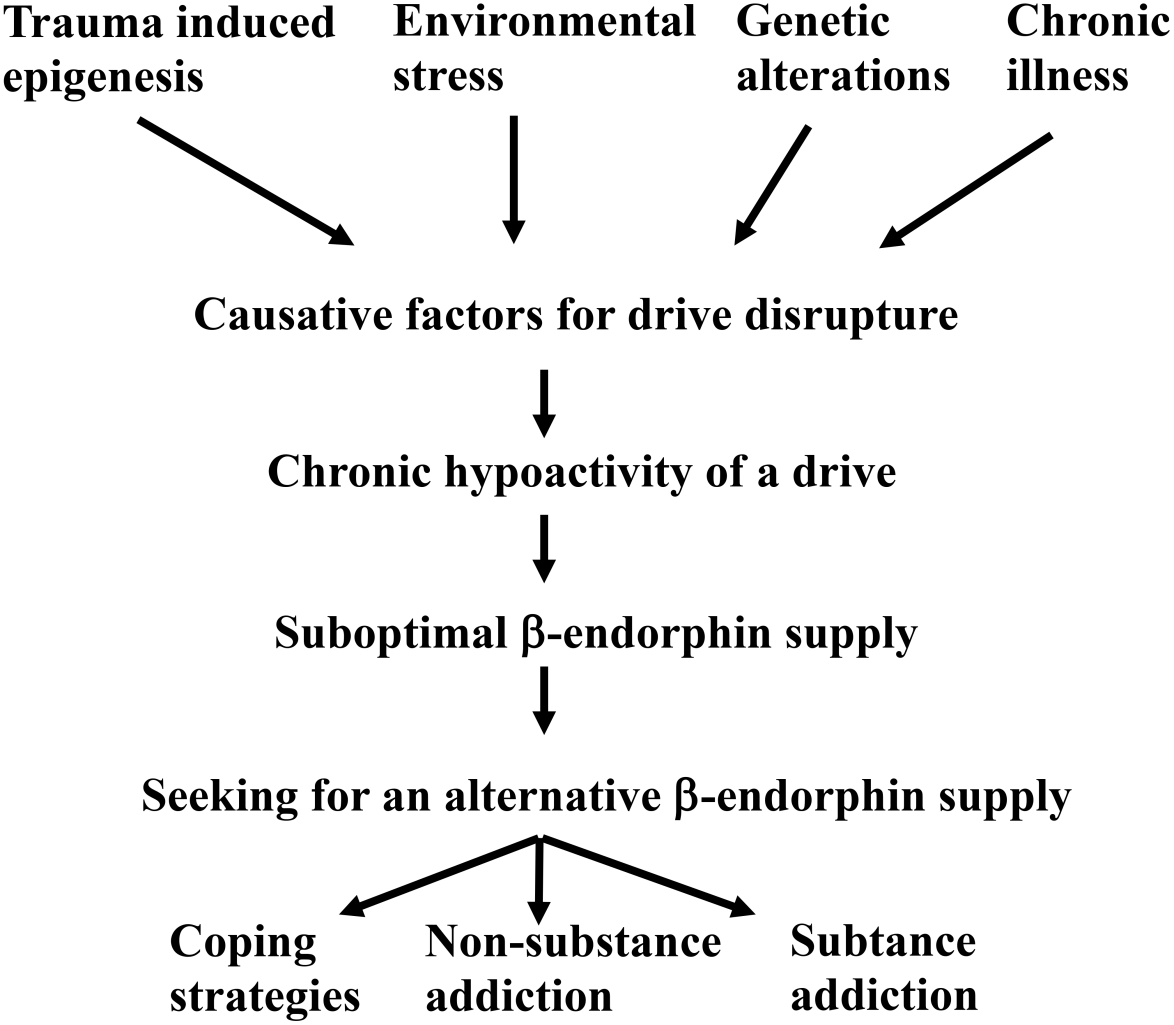
Onset of an addiction disease via a malfunctioning Freudian drive.

In summary, a causative factor can lead to the malfunctioning of an essential drive (**Scheme 5**).

As a result of the hypoactive drive, the patient is unable to maintain physiological β-endorphin levels and seeks an addiction – whether coping strategies, non-substance use or substance use addiction - to compensate for the deficit.

Addiction often co-occurs with major psychiatric disorders. Ricci et al. (183, 184) showed that users of synthetic cannabinoids experience more severe psychotic symptoms, higher aberrant salience, dissociation, and suicidal ideation than non-users or natural cannabis users.

These findings highlight the need to both view addiction as a causative factor for other disorders and to critically deconstruct the concept of addiction itself.

### 6.2 Possible therapy options

From Freud´s (somewhat hidden) assertion that the functioning of all five essential drives counteracts the resilience of addiction (*vide supra*), an important therapeutic goal for treating addiction should be the revitalization of the disrupted drive. Consistently, the author was unable to identify an instance of addition coexisting with all five drives functioning healthily.

Given that β-endorphin levels are decreased in various addictions (**Table 1**), addiction could be classified, like other conditions (e.g., diabetes mellitus, hypothyroidism, morbus Parkinson) as a deficiency disease of a signal molecule. For patients with such disorders, hormone replacement therapy is often the treatment of choice. Indeed, laboratory studies have demonstrated that cocaine addiction can be successfully treated with exogenous β-endorphin (178). However, clinical applications of exogenous β-endorphin in psychiatric patients have been limited due to ethical concerns, as β-endorphin, like heroin and morphine, is a μ-receptor agonist (142, 143) and thus carries a potential for addiction. Preliminary investigations into β-endorphin treatment were reported in four bipolar and two unipolar depressive female patients (185). Bipolar disorder with depression is associated with both very low β-endorphin levels (**Table 1**, Entry 19) and a 48% prevalence of addiction (non-substance and substance misuse) (186). While β-endorphin injections induced a noticeable improvement in depressive psychopathology within 20 - 30 minutes, the effect generally lasted only two hours, with four out of six patients relapsing spontaneously (185). As a result, the high addiction potential and short duration of exogenous β-endorphin´s therapeutic effect prelude its use as a reliable hormone replacement therapy.

Since hormone replacement therapy cannot be effectively implemented, the use of selective serotonin reuptake inhibitors might be considered. Serotonin (5-hydroxytryptamine) plays a role in inducing β-endorphin release (**Schemes 2**, **3**), and studies have shown that fluoxetine significantly increases β-endorphin levels in the *arcuate nucleus* and *nucleus accumbens* in laboratory animals (187).

As fluoxetine, like the drugs listed in **Table 6**, releases β-endorphin in the *nucleus accumbens*, it may reduce the capacity of these drugs to induce satisfaction, thereby decreasing the patient´s need for their consumption. In fact, a meta-analysis of 64 randomized controlled trials involving 6128 participants demonstrated that fluoxetine facilitated abstinence from opioids, alcohol, cocaine, cannabis, and nicotine while also reducing depressive symptoms (188). However. it should be noted that serotonin acts as a termination signal for the Freudian drives outlined in **Scheme 2** and **Table 7**. Prolonged use of serotonin reuptake inhibitors may suppress these drives, potentially rendering the quality of life for patients. Consistent with these findings, while fluoxetine is approved in the United States for treating depression in children and adolescents (189), there remains uncertainty about whether selective serotonin reuptake inhibitors are associated with an increased risk of suicidal ideation and behavior in individuals under 25 years of age (190). Given these uncertainties, the use of fluoxetine in patients under 25 years old should be considered a treatment of last resort. Since major suicide repeaters exhibit elevated β-endorphin levels (**Table 1**, Entry 4), and the role of this opioid during such life-threatening acts remains poorly understood, the SSRI-induced increase in synaptic serotonin activity may represent a contraindication in suicidal patients.

Among these, the *HTR1A* gene — encoding the 5-HT_1A_ receptor — is of particular clinical importance. In psychiatric patients, epigenetic modification of *HTR1A* (see **Table 4**) can be identified by increased methylation, which is measurable in leukocyte DNA (114, 115). Such hypermethylation leads to reduced postsynaptic 5-HT_1A_ receptor expression and diminished serotonin-induced β-endorphin release. Consequently, SSRIs, which rely on intact 5-HT_1A_ signaling (see **Scheme 3**), may show limited therapeutic efficacy in such patients.

From the perspective of the updated Freudian drive theory, it may therefore be clinically prudent to evaluate the epigenetic status of the *HTR1A* gene *before* initiating SSRI treatment with agents such as fluoxetine.

In addition, psychological therapies may have the potential to revitalize the disrupted drive (**Table 8**).

**Table 8:** Evidence-based psychological treatments for substance addiction.

Method	Description	Empirical evidence
Cognitive Behavioral Therapy (CBT)	Targets maladaptive thoughts and behaviors; develops coping skills to reduce substance use.	Meta-analyses [e.g. (191, 192)] show moderate to strong efficacy for substance use disorders.
Motivational Interviewing (MI)	A client-centered approach to enhance intrinsic motivation by resolving ambivalence.	Broadly supported across substances; highly effective in early-stage treatment (193).
Motivational Enhancement Therapy (MET)	A structured version of MI that integrates assessment feedback to promote behavioral change	Shown to reduce alcohol and cannabis use in randomized trials (194).
Relapse Prevention (RP)	Teaches clients to anticipate and cope with high-risk situations to prevent relapse	Effective in maintaining abstinence post-treatment (195).
Contingency Management (CM)	Uses tangible rewards (e.g. vouchers, privileges) to reinforce abstinence or treatment goals.	Strong empirical support, especially in stimulant and opioid use disorders (196).
Community Reinforcement and Family training (CRAFT)	Combines behavioral strategies and family involvement to engage unmotivated individuals in treatment.	Demonstrated higher engagement rates than traditional interventions (197).
Acceptance and Commitment Therapy (ACT)	A third-wave CBT approach focused on acceptance, mindfulness, and values-driven action.	RCTs support its efficacy across multiple addiction types (198).
Mindfulness-Oriented Recovery Enhancement (MORE)	Integrates mindfulness, reappraisal, and savoring to reduce addictive behavior and improve emotional regulation.	Superior to CBT in some trials for pain-related opioid misuse (199).

All listed methods are supported by peer-reviewed randomized controlled trials (RCTs) or meta-analyses. CBT, MI/MET, and RP are considered core treatment; CM, CRAFT, ACT, and MORE offer complementary or population-specific benefits.

However, many psychoanalysts would agree that persistent non-substance addictions (e.g., masochism, pathological gambling, anorexia nervosa, and particularly bipolar disorders) often show limited responsiveness to individual psychotherapy. Notably, nearly all of these disorders involve the corruption of the attachment drive. Thus, psychotherapeutic interventions should focus on revitalizing the impaired attachment drive. This can be achieved by emphasizing interpersonal orientation and incorporating group psychotherapy into treatment plans.

### 6.3 Limitations

1. To avoid delving into the labyrinth of biochemical possibilities, only well-analysed signal molecules, such as β-endorphin, were proposed as having key importance in the processing of both essential drives and addictions. While this approach aligns with current knowledge, my conclusions may require revision if presently underexplored metabolites or under-evaluated brain areas are later found to influence the suggested signal-molecules.
2. As noted by an anonymous reviewer (see above), the Bowlbyian drive (attachment) is less well supported by experimental data compared to the other drives. Although I have expanded the connection to experimental findings in the revised version — by introducing relevant receptors and discussing the potential impact of epigenetic changes on both attachment and β-endorphin activity (see **Table 4**, **Scheme 3**) — the deconstruction of this drive remains primarily limited to the mother-infant bond from the infant’s perspective. As such, this framework may help explain potential consequences of (early) childhood trauma but does not provide reliable predictions for psychological trauma occurring in adulthood.
3. Although I believe I have identified nearly all relevant studies on the role of β-endorphin in addiction, the number of available studies is limited, and randomized controlled trials are notably scarce. As a result, it can be confidently stated that Freudian drive disruption can induce addiction, but it cannot be definitively concluded that such a disruption is a necessary prerequisite for addiction.

### 6.4 General conclusion

Give the addicted patient five: five healthy drives!

## Data Availability

The original contributions presented in the study are included in the article/supplementary material. Further inquiries can be directed to the corresponding author.

## Footnotes

^1^
Freud distinguished in his work between drives (*Triebe* in German) and instincts (*Instinke* in German). As mentioned in the abstract, both terms were unfortunately translated as “instincts”, introducing unnecessary ambiguities. Recently, the precise distinctions between drives, instincts, and reflexes have been clarified (11).

^2^
For example, the motor factor of the hunger drive, i.e., the hormone ghrelin, was identified 60 years after Freud´s death. (14)

^3^
The term “reward” was introduced by Skinner (19) in psychology and was later applied to rat behaviour (20), whereas Freud´s overlooked yet compatible term, “satisfaction”, is used exclusively for human beings. Since rats cannot experience psychotic illness, the author considers “satisfaction” to be the more appropriate term.

4 Referring to the deconstruction of the attachment drive proposed by (55), an anonymous reviewer noted:*“Although intriguing, the 'Bowlbyian drive' theory is not as well supported by empirical data as the other drives.”* Unfortunately, this observation is valid; however, an updated version of the deconstruction is now possible, presenting key receptors that may be modified through childhood trauma–dependent epigenesis (*vide infra*).
